# Emotional facial perception development in 7, 9 and 11 year-old children: The emergence of a silent eye-tracked emotional other-race effect

**DOI:** 10.1371/journal.pone.0233008

**Published:** 2020-05-11

**Authors:** Jennifer Malsert, Amaya Palama, Edouard Gentaz

**Affiliations:** 1 SensoriMotor, Affective and Social Development Laboratory, Faculty of Psychology and Educational Sciences, University of Geneva, Geneva, Switzerland; 2 Swiss Center for Affective Sciences, Campus Biotech, University of Geneva, Geneva, Switzerland; 3 University of Teacher Education, Special Needs Education Unit, State of Vaud (HEP Vaud), Lausanne, Switzerland; 4 University of Grenoble Alpes, LPNC, CNRS, Grenoble, France; Bournemouth University, UNITED KINGDOM

## Abstract

The present study examined emotional facial perception (happy and angry) in 7, 9 and 11-year-old children from Caucasian and multicultural environments with an offset task for two ethnic groups of faces (Asian and Caucasian). In this task, participants were required to respond to a dynamic facial expression video when they believed that the first emotion presented had disappeared. Moreover, using an eye-tracker, we evaluated the ocular behavior pattern used to process these different faces. The analyses of reaction times do not show an emotional other-race effect (i.e., a facility in discriminating own-race faces over to other-race ones) in Caucasian children for Caucasian *vs*. Asian faces through offset times, but an effect of emotional face appeared in the oldest children. Furthermore, an eye-tracked ocular emotion and race-effect relative to processing strategies is observed and evolves between age 7 and 11. This study strengthens the interest in advancing an eye-tracking study in developmental and emotional processing studies, showing that even a “silent” effect should be detected and shrewdly analyzed through an objective means.

## Introduction

Emotional facial perception is a complex process that develops in childhood from the earliest days of life. The ability to recognize facial expressions emerges early, at around the age of 7 months [1–3]. Despite this early emotional skills enhancement, the improvement of facial expression perception persists during childhood until about age 14 [4] parallel to frontal cortical maturation. Kolb, Wilson and Taylor [4] showed that happy faces were perceived equally well by 6-year-old children as by adults whereas other emotional faces were perceived poorly until adolescence, showing the importance of considering the development of emotional facial expressions individually. Thus, these recognition abilities improve considerably with age throughout childhood and (pre)adolescence [5, 6]. Based on several different tasks, studies provide a consensus regarding the developmental trajectory of facial expression recognition: overall, 4- to 5-year-old children performed as well as 6– to 9-year-olds for happiness, but not for sadness, anger, or fear, respectively to chronology [7–9]. Surprise and disgust are recognized later, between ages 6 and 10 [10]. Nevertheless, this consensus is debatable due to the diversity of tasks [6, 11–13]. Therefore, with age, children should increase their expertise in facial expression processing through automation in emotional interpretation [14].

In addition to emotional information which may enrich face processing, the ethnicity of the face should interfere with discrimination. Indeed, it is well established in literature that other-race faces are more difficult to recognize than own-race ones through the so-called Other-Race Effect (ORE) phenomenon [15]. Different ethnic groups have shown this robust effect with faster and more accurate recognition of own-race faces [16–24]. This narrowing for own-race face processing should emerge early, during the first year, even as of the third month of life [25–33]. Nevertheless, the specialization of face processing depends on early visual experience with ORE actually more related to the ethnicity of the child’s main caregivers than to the child’s own [34]. Accordingly, children with parents of different ethnicities or those who are heavily exposed to more than one ethnic group develop expertise for more than one group [28, 35, 36]. Based on cerebral plasticity, familiarization or training to faces from a new ethnic group allows children to maintain abilities for other races [19, 37, 38] and this flexibility has been observed in 3- to 14-year-old adopted children [20, 39]. Valentine [24] presented the face expertise development as a framework in terms of vectors in a multidimensional perceptual space in which an average of all our experienced faces would compose a ‘prototypical face’. Thus, faces are encoded as vectors according to their deviation from this prototypical face. Predominant exposure to faces of a specific species, gender, or race early in life will cause the dimensions of one’s prototype to become “tuned” towards such faces. Faces close to this ‘prototype’ are thus easily categorized [40]. Caroo [41, 42] had already observed this environmental facilitation through a significant own-race advantage in recognition, and a positive effect of interracial experience for recognition of other-race faces.

As for their discrimination, ethnic familiarity increases processing of the emotional expressions of faces [43–47]. In their meta-analysis, Elfenbein and Ambady [48] showed that emotions were universally recognized above chance level but that there was a pervasive in-group facilitation. This experience-dependent advantage would decrease when participants lived in more ethnically diverse regions or reported out-group experiences [49]. Moreover, ethnic group would impact emotional processing itself, and children across cultures may display distinct patterns of socio-emotional functioning in early childhood. For example, Chinese children displayed more self-regulation in compliance and delay tasks than North American children did [50–52]. Cross-cultural differences have been demonstrated in social interaction and may affect children’s emotional sensitivity and course of development. In adults, this cultural particularity has been observed by Ishii and collaborators [53] using an offset task [54]. Participants were required to respond to a dynamic facial expression video when they believed that the first emotion presented had disappeared. Compared to US subjects, Japanese adults perceived the offset of happiness faster than Americans did. Authors interpreted this sensitivity to the disappearance of positive emotional expression as related to an Asian cultural anxiety and sensitivity to others’ expectations [50–52, 55, 56].

Less is known about how faces are processed depending on different developmental, emotional or ethnic factors. However, eye-trackers could constitute a major source of information in facial and emotional processing. Few studies have been interested in ocular behavior to faces, and even less with cultural or emotional factors. Eye tracker studies during childhood have shown patterns of preferential fixations according to emotional expression, distributed over internal features such as eyes and mouth [57]. For example, Dollion and collaborators [57] showed that infants looked preferentially at the mouth for happy face processing, whereas they oriented toward the eyes and eyebrow areas for angry and sad faces. Thus, it was suggested that our predisposition for face processing also differs across cultures for the strategy employed to extract visual information from faces [58]. Among the rare cultural eye-tracking studies, a difference in fixation pattern between Caucasian and East Asian people emerged. For example, Liu and collaborators [59] demonstrated that Asian infants (4 to 9 months old) predominantly look around the nose, avoiding a direct gaze toward the eyes. By contrast, Caucasian infants (6 to 10 months old) preferentially look at the eye area to process faces [60]. In adults, Blais and collaborators [58] have shown that, to categorize and recognize faces, Caucasians looked at the eye and mouth areas whereas Asians used mostly the central area, around the nose. These data were consistent with a theory supported previously by Kitayama and collaborators [61], suggesting a principally holistic processing for faces in Asian adults compared to an analytic one in Caucasian adults. Surprisingly, an eye-tracked study [62, 63] revealed that, even though the environment modulates expertise for the types of faces experienced, this social experience does not abolish cultural diversity in eye movement strategies.

In this study, we aim to analyze reaction times and/or ocular behavior to evaluate whether the development of face processing depending on emotional and ethnic faces can be observed in children between 7 and 11 years old, an age sensitive to emotional understanding enhancement. For this aim, we choose to use an emotional offset task as developed by Niedenthal et al. [54], which has already been shown to be adaptable to ethnic effects studies [53]. In a previous Offset study with Asian and Caucasian faces, the analyses of reaction times revealed a clear emotional ORE in Vietnamese children but not in Swiss ‘Multicultural’ children, demonstrating the importance of an integrated environment in face processing [64]. In the present study, we examined if a larger Swiss Caucasian child population would be sensitive to ethnicity of faces through an emotional ORE and to emotion through a developmental course performance between happy and angry expressions. Based on previous study results, we do not expect facilitation in reaction times in this integrated population, but suggest that a developmental emotional effect could be demonstrated in angry face processing, due to the developmental course for this emotion.

Using an eye-tracker, we evaluate the ocular behavior pattern used between ages 7 and 11 to process these different faces. The ocular movement analysis could provide more finer data depending on the face ethnicity and emotion processed. Happy faces would be processed more easily in younger children parallel to emotional understanding development in the age range. If analytic fixation patterns are already developed, we could observe interaction between face and area of interest, i.e. the mouth and zygomaticus for happy faces and the eyes and corrugators for angry faces.

## Materials and methods

### Participants

A total of 88 children (48 girls 40 boys) aged 7, 9 and 11 years were recruited in 3 Swiss multicultural public schools in the canton of Geneva and participated in the present experiment (Table 1). Parents had previously signed an informed consent and completed a Socio-Economic Status questionnaire and questions about cultural origin and familial environment. Children’s birth date and term, country of birth, residence or education since birth, mother tongue and ethnic types present in the child’s immediate environment (Asian, Caucasian and/or other) were controlled. Children who were themselves, or who lived or had lived in an environment that was neither Asian nor Caucasian were included in a so-called "multicultural" group. The questionnaire indicated 39 Caucasian, and 49 ‘Multicultural’ environments, i.e. neither Caucasian nor Asian children. Seven subjects were excluded from the looking times analysis due to the loss of eye detection by the eye-tracker or recording failures. Thus, 81 children were analyzed for eye-tracking looking times (46 girls of whom 23 are Caucasian, 35 boys—14 Caucasian).

**Table 1:** Participants repartition for reaction times and eye-tracking analysis.

Reaction times				Eye-tracking looking times			
Age Group *(Mean±SD)*	Environment	N	Total *(N girls)*	Age Group *(Mean±SD)*	Environment	N	Total *(N girls)*
7 y.o *(6*.*68±0*.*48)*	Caucasian	13	25 *(14)*	7 y.o *(6*.*65±0*.*49)*	Caucasian	12	20 *(12)*
	Multicultural	12			Multicultural	8	
9 y.o *(8*.*58±0*.*50)*	Caucasian	11	25 *(14)*	9 y.o. *(8*.*61±0*.*50)*	Caucasian	10	23 *(14)*
	Multicultural	14			Multicultural	13	
11 y.o. *(10*.*50±0*.*62)*	Caucasian	15	38 *(20)*	11 y.o. *(10*.*50±0*.*62)*	Caucasian	15	38 *(20)*
	Multicultural	23			Multicultural	23	
**Total**			**88**	**Total**			**81**

This study was carried out in accordance with the latest revision of the World Medical Association’s Code of Ethics (Declaration of Helsinki), and was approved by the Institutional Review Board at the University of Geneva (Commission d’Ethique FAPSE).

### Stimuli and procedure

The stimuli presented consisted of 5-second video clips showing gradual changes in emotional expressions displayed by adult faces in frontal pose [64]. Caucasian face stimuli were constructed from 20 adult face pictures (50% female) selected and standardized black and white pictures of 5°×6.8° of visual angle. The same photos have been successfully used to create similar stimuli in previous studies [54, 65–67]. Asian face stimuli were constructed in the same way with 20 adult faces (50% female) selected from the Asian Emotion Database [68, 69]. Asian database and pictures were selected in order to correspond as possible to Caucasian faces in terms of size, resolution, contrast and luminance, thus converted in black and white. To create morphed images depicting the continuum between two faces (pictures of a person with happy and angry expressions), the positions of the features in one photograph are moved toward their positions in the other photograph, as if the image lay on a rubber sheet. Each angry- and happy-face picture was paired and morphed progressively from one emotion to the other with a software (Morpheus Photo Morpher, version 3.17) producing 500 frames by paired stimuli, converted afterwards into movies at 100 frames per second with ffmpeg (http://ffmpeg.org/) [66, 67]. The 5-second movie clips always showed a full-blown expression of happiness or anger that gradually morphed into the other expression. Four blocks (2 Asian and 2 Caucasian faces counterbalanced) of 20 trials were performed and separated by a short break.

The experiment took place in a quiet room at the children’s school where they were tested individually with a computer task. Eye movements were recorded with an eye-tracker SMI RED 250 (SensoMotoric Instruments GmbH, Teltow, Germany). The experiment started with a 9-point calibration phase at different locations covering the whole surface of the screen. This phase was repeated until a satisfactory calibration (less than 2° of deviation in the x and y axes) was achieved. Video clips of adult faces showing one emotion (e.g. Happiness) moving towards another (e.g. Anger) were presented to each child. The children were instructed to report when they no longer perceived the initial first emotional expression by pressing the “Space” button of the computer with their writing hand’s index finger. Each child completed 80 trials divided into 4 blocks for a total of 12 to 15 minutes.

### Data analyses

**Fig 1:**
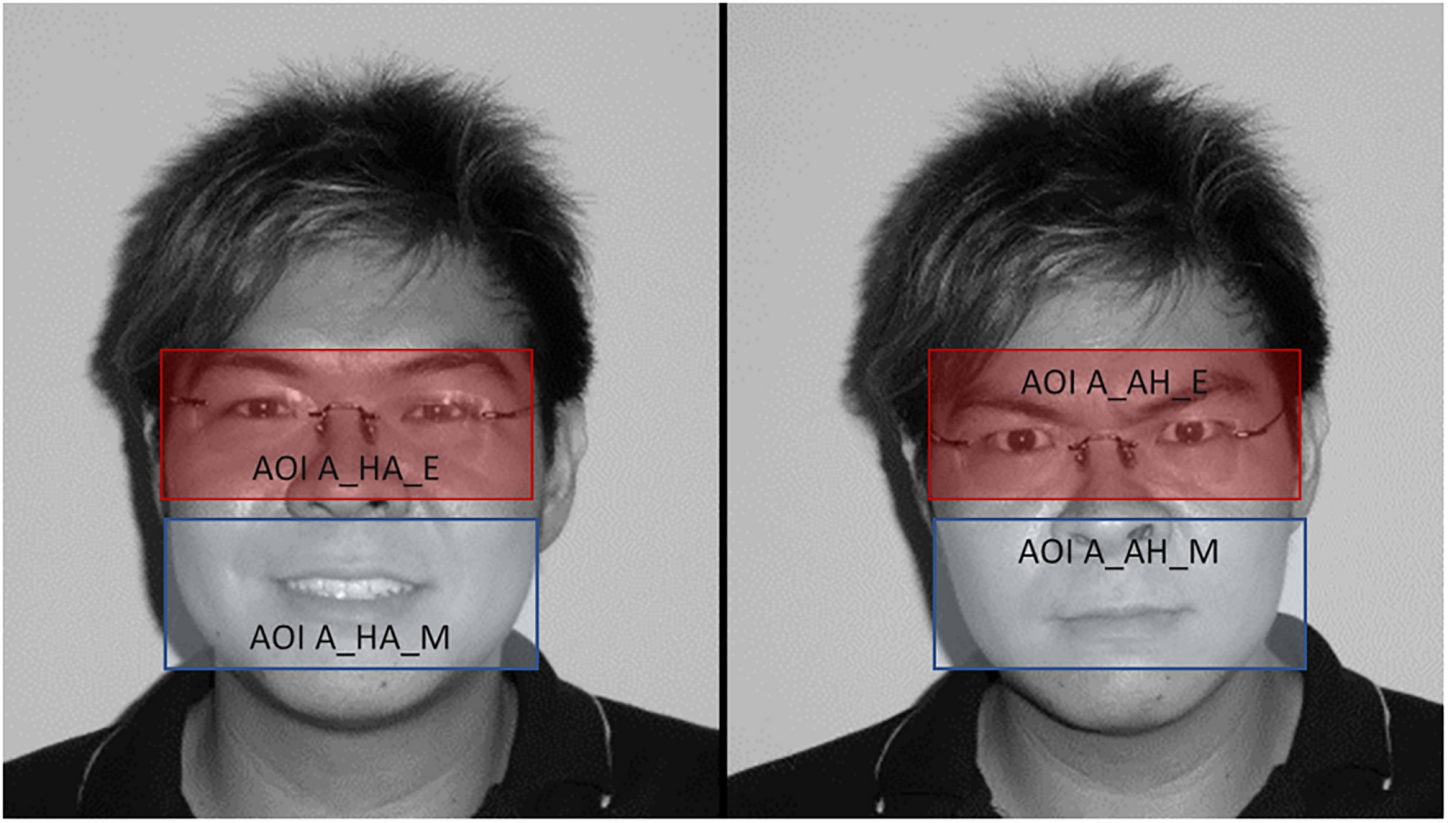
Scene captures of video for both emotions, overlaid with areas of interest (AOIs) for the eyes and mouth regions. This individual (subject 27 from Asian Emotion Database) in this figure has given written informed consent to publish these images.

Analyses were interested in 1) behavioral offset reaction times to press the button for the emotional offset evaluation; 2) eye-tracking recorded mean looking times toward specific areas of the faces (Area Of Interest, AOI). AOIs have been delineated on the basis Ekman and Friesen’s Facial Acting Coding System [63] in which the contractions or decontractions of the face are broken down into action units. We used action units to the emotional expression of happiness and anger, and defined ‘universal’ AOIs that allow us to cover the entire eye or mouth areas for each face regardless of moves associated to emotional expression in movies as in Berdasco-Muñoz, Nazzi and Yeung [70]. In this view, the area called ‘eyes AOI’ contained the orbicular muscles of the eye for the cheek lift specific to joy (UA6) as well as the tension of the eyelid and the opening between the upper eyelid and the eyebrows specific to anger (UA5 and UA7). The ‘Mouth AOI’ included the orbicular muscle of the mouth activated in the closing tension of the lips proper to the expression of anger (respectively UA4 and UA23). The area of the mouth also included the muscle of the great zygomatic used to stretch the corner of the lips to make them smile (UA12). All AOIs are identical to those shown in Fig 1 and have a size of 1.2 x 3.5°. These ‘Eyes’ and ‘Mouth’ extended areas allowed us to analyze the videos with a constant in the AOI as demonstrated in Berdasco-Muñoz, Nazzi and Yeung [70]. Looking times were extracted from the SMI program through Net Dwell Times for the first half of each video morph timeline (2500 ms) in order to evaluate ocular movement for the first emotion offset evaluation corresponding to response decision making.

Afterwards, reaction and looking times greater than two standard deviations from the mean were not considered for analysis (<2%). Statistical analyses were conducted using Statistica 13. The significance threshold was .05 and planned contrasts were performed to contrast significant interactions. Effect sizes are given in partial eta-squared η_p_^2^ for ANOVAs.

## Results

### Offset reaction times

A Stimulus Face ethnicity (Asian/Caucasian) x Emotion (Angry to Happy / Happy to Angry) repeated measure ANOVA was performed on mean RTs for the 88 children with Age Group (7, 9, 11) and Participant Environment (Caucasian, Multicultural) as between-subject factors.

#### Stimuli race effect and emotion effect

**Fig 2:**
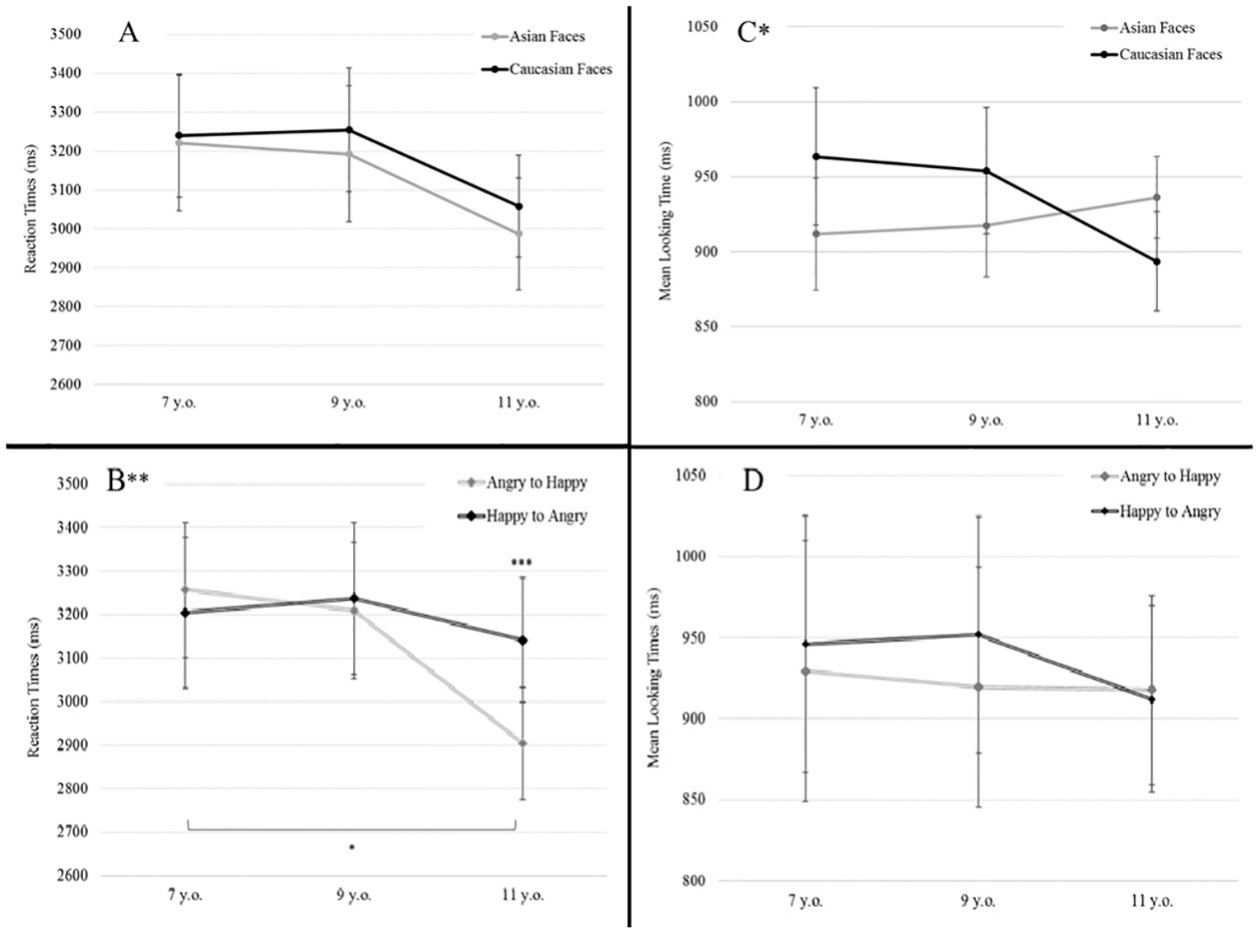
**A.** Non-Significant Face ethnicity * Age Group and **B.** Significant Emotion * Age Group Interactions in Offset Reaction Times (RT). **C.** Significant Face ethnicity * Age Group; and **D.** Non-Significant Emotion * Age Group Interactions in Mean Looking Times. *: p<.05; **: p<.01; ***: p<.001.

There is no main effect of Stimulus Face ethnicity (*F*(1,82) = 1.44, *p* = .234, η_p_^2^ = .017), interactions with Age Group (*F*(1,82) = 0.14, *p* = .867, η_p_^2^ = .003) (Fig 2A) or Participant Environment (*F*(1,82) = 0.96, *p* = .33, η_p_^2^ = 0.012).

There is a trend for main effect of Emotion (*F*(1,82) = 3.54, *p* = .063, η_p_^2^ = .041) but a significant interaction between Emotion and Age Group is observed (Fig 2B). Indeed, Angry offset reaction times decrease with age more than Happy expression (*F*(2,82) = 5.87, *p* = .0042, η_p_^2^ = .125).

Contrasts from planned comparison show that offset perception is significantly shorter for Angry faces than for Happy ones in the 11 year-old group only (2905 *vs*. 3141 ms, *F*(1,82) = 17.25, *p*<.0001). Moreover, oldest children have decreased Reaction Times than youngest ones for the Angry to Happy expression (2905 *vs*. 3257 ms, *F*(1,82) = 6.14, *p* = .015).

#### Emotional race effect

An interaction between all factors, Face Ethnicity, Emotion, Age Group and Environment is also observed (*F*(2,82) = 3.2, *p* = .046, η_p_^2^ = .072). Contrast analysis shows the significant effect of emotion for 10–12 year-old children, with decreased RT for Anger offset compared to Happiness offset particularly for Caucasian Environment children processing Caucasian faces (2782 *vs*. 3225 ms, *F*(1,82) = 15.03, *p*<.001), but not for Multicultural children (3056 vs. 3171, *F*(1,82) = 1.55, *p* = .217) or in Caucasian environment children for Asian Faces (2840 vs. 3017, *F*(1,82) = 2.44, *p* = .122).

### Eye tracker looking times

Looking times were analyzed on the first half of the videos in order to observe ocular behavior 1) depending on the first emotional expression of the morph; and 2) before motor response. The significance threshold was .05; effect sizes are given in partial eta-squared η_p_^2^ for ANOVAs main effects. Planned comparisons were performed for significant interaction contrasts.

### Mean looking times

Stimulus Face ethnicity (Asian/Caucasian)] x Emotion (Angry to Happy / Happy to Angry) x AOI (Mouth/Eyes) repeated measures ANOVA with Age Group (7, 9, 11) and Participant Environment (Caucasian, Multicultural) as between-subject factors were performed on mean Looking Times on the first half of morphs for the 81 children.

We observe a main effect of AOI; mouth areas are more watched than eyes (1047 *vs*. 812 ms, *F*(1,75) = 5.37, *p* = .023, η_p_^2^ = .067) and different interactions are demonstrated.

#### Race effect

First, Face ethnicity interacts with children’s Age group (Fig 2C), with an inversion of looking time between Caucasian and Asian faces, appearing in the older group, with faster Caucasian face processing in 10–12 year-old children (*F*(2,75) = 3.17, *p* = .048, η_p_^2^ = .078).

The second interaction concerns Face ethnicity and AOI (*F*(2,75) = 16.32, *p* = .0001, η_p_^2^ = 0.178), in which we observed a significant difference between mouth and eye looking times for Caucasian face processing (1123 vs. 751 ms, *F*(1,75) = 12.39, *p* = .0007), not observed for Asian faces (972 vs. 872 ms, *F*(1,75) = 0.84, *p* = .3618). There is no main effect of children’s environment (*F*(1,75) = 2.58, *p* = .362, η_p_^2^ = .033).

#### Emotion effect

There is no main effect of emotion in mean looking times (*F*(1,75) = 1.33, *p* = .253, η_p_^2^ = .017) nor interaction between Emotion and Age group (Fig 2D) (*F*(1,75) = 0.95, *p* = .391, η_p_^2^ = .024).

#### Emotional-race effect

**Fig 3:**
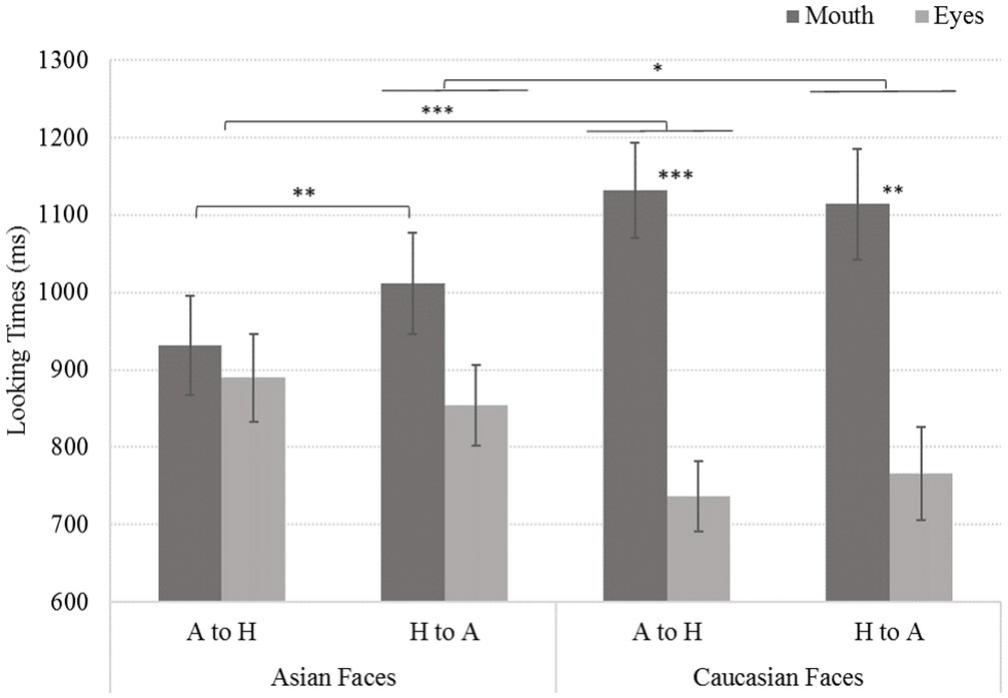
Looking time interaction for face ethnicity, emotion and area of interest (AOI). A to H: Angry to Happy, H to A: Happy to Angry. *: p<.05; **: p<.01; ***: p<.001.

The last interaction involves Face ethnicity, Emotion and AOI (Fig 3) (*F*(1,75) = 5.68, *p* = .019, η_p_^2^ = .070). This interaction shows that the Face ethnicity effect is observed particularly for Anger offset evaluation, with a significant difference in AOI looking times (Fig 3). Children looked more at the Mouth for Caucasian faces than for Asian ones (1132 vs. 931 ms, *p*<.0001) and at the Eyes for Asian faces compared to Caucasian ones (890 vs. 763 ms, *p*<.0001).

Moreover, the processing of Caucasian faces shows a significant difference between Mouth and Eyes AOIs for both emotions (Angry: 1132 vs. 736 ms, *F*(1,75) = 17.03, *p*<.001; Happy: 1114 vs. 766 ms, F(1,75) = 1, *p* = .006).

However, Asian emotional expressions do not differ between AOI looking times, but the Happy expression showed longer looking times toward the mouth than the Angry one (1012 vs. 931 ms, *F*(1,75) = 7.63, *p* = .007).

### Percentage between AOI looking times

**Fig 4:**
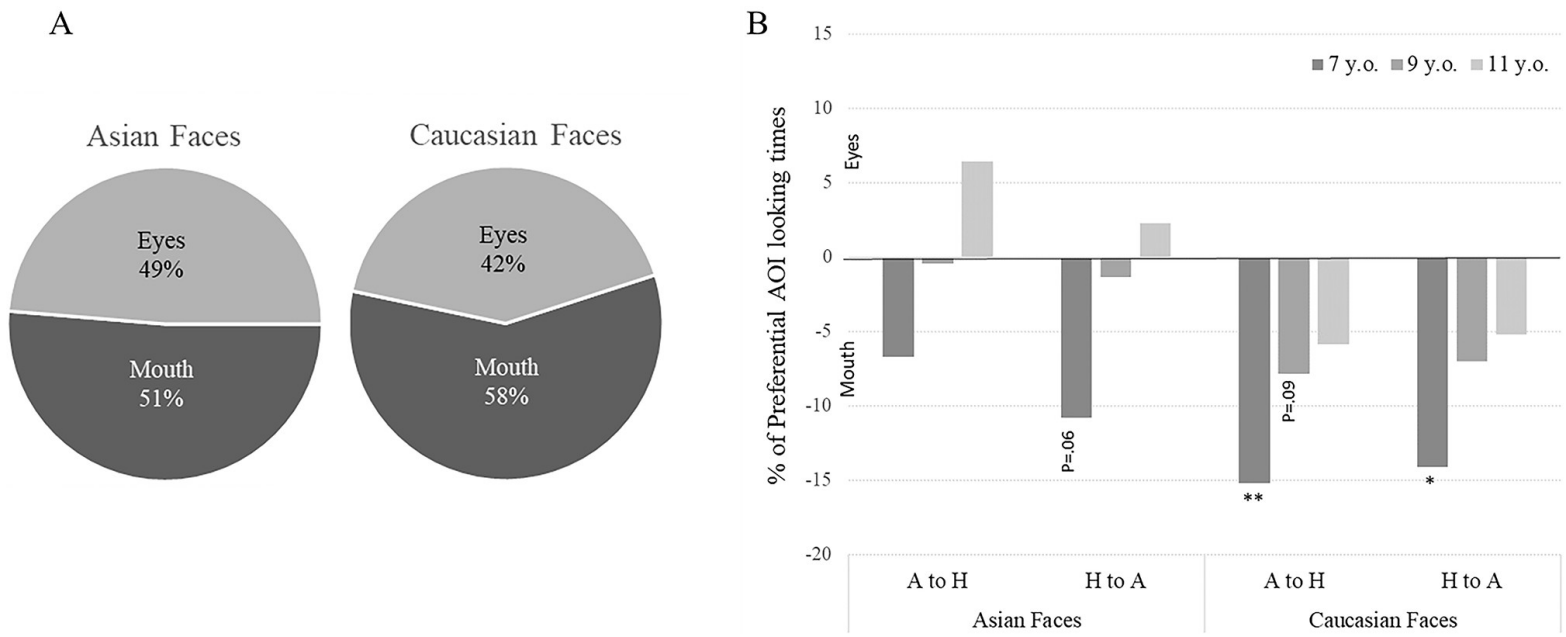
**A.** Percentage of looking time between mouth and eyes AOI depending on Faces. **B.** Percentage of preferential AOI looking times depending on Age, Faces and Emotion. *:p<.05; **: p<.01.

A second rmANOVA analysis was completed on AOI looking times percentages in order to see the proportion of looking time between each AOI. We observe an effect of Face (F(1,75) = 15, p<.001, ηp2 = .166). Asian faces were treated equally between mouth and eyes areas, whereas Caucasian were more scanned from mouth (Fig 4A).

The average percentage of looking times to each AOI was calculated across conditions and compared with the percentage expected by chance (.50) using two-tailed t-tests. Results were presented in Fig 4B as percentage of preferential AOI looking times (% Eyes AOI–expected by chance .50). A positive percentage therefore corresponds to a preference for Eyes AOI, while a negative percentage corresponds to a preferential orientation towards the mouth AOI. Results showed that 7-year-old children preferentially looked at mouth AOI to process Caucasian Faces Angry (-15,2%, p = .005) and Happy (14,1%, p = .042) expressions whereas no preferential orientation was found for Asian Faces.

## Discussion

This study aimed at evaluating the developmental impact in emotional expression discrimination depending on the ethnicity of faces. For this aim, we included 7, 9 and 11 year-old children in a multicultural public school who had to perform an offset task, in order to detect their sensitivity to facial emotional cues through reaction times and/or ocular behavior.

There was not a significant difference in reaction time performance between Caucasian and Asian emotional expression processing. This result is consistent with a previous study using this task that showed an emotional other-race effect in Vietnamese children but not in their Swiss counterparts [64]. This observation is made in the integrated Swiss population, in a city composed of 48% foreign residents [71, 72]. The ability to discriminate emotions would be more generalized in people living in an environment with heterogeneous ethnicities, as previously suggested in adults by a meta-analysis published by Elfenbein and Ambady [48]. Nevertheless, reaction times show that, even if the Happy expression evaluation does not evolve between Age groups, Anger offset detection becomes shorter in the older group. This observation is consistent with emotional development, with a stabilized processing of Happiness early in development but a later development of the understanding in more complex and less experienced emotions such as Anger, which is known to be efficiently processed between ages 8 and 12 [6].

Eye-tracker data were analyzed to deal with these observations in depth. We found here that gaze behaviors towards emotional faces showed several differences depending on Age, Emotion or Face ethnicity. A main impact should be the effect of Face ethnicity processing through the age groups. Indeed, we observe a decrease in Caucasian face processing times with age, suggesting the emergence of an eye-tracked emotional Other-Race Effect in children despite its absence in offset reaction times. The Caucasian faces should also be more processed by AOI, with longer looking times around the mouth area than the eyes area, which was not observed for Asian faces. Asian faces processing was more oriented toward the mouth area for the Happy evaluation than for the Angry evaluation. This observation made on net looking times was supported by the results of the analysis of the percentage of AOI preferential looking times. This finding could be consistent with Kitayama and collaborators’ study [61], which showed a preferential focal feature strategy used by Caucasians to process faces, whereas Asian people used more holistic and global strategies, as supported by the findings of Blais and collaborators [58]. Here, it was the Caucasian faces that were more analyzed by preferential features compared to Asian faces treated more centrally, even by the same population. However, we also see that with age, Caucasian faces tend to be processed more equitably among AOIs. Interpretation in holistic treatment must therefore be approached with caution as it may involve the development of better processing skills for the ocular region. Indeed, youngest children show a better early use of information from the mouth, associated to earlier processing skills of happy expressions.

Altogether, our results show a developmental course for emotional facial expression, with the later improvement of Anger processing compared to the already well-established happy face processing, consistent with the early development of happy emotion preference and understanding [4–6]. It has been previously shown that happiness is correctly conceptualized as of age 6, with efficient component processing. Indeed, happiness is not sensitive to the inversion effect avoiding holistic treatment [73], thus, the mouth as an analytic component for smile processing could be enough [74]. Eye-tracking data corroborate the preferential orientation towards the mouth area to process happiness. On the other hand, anger did not orient children preferentially toward the eye area and specific activations of the corrugator, but we saw through reaction times that anger processing is under development at this period. Angry faces could require a holistic processing which could develop gradually during childhood [57, 58, 61, 75, 76], or a better Eyes AOI orienting to process corrugator areas. Nevertheless, our results show that, more than an Asian *vs*. Caucasian people interaction strategy, Kitayama’s results with Asian holistic *vs*. Caucasian analytic processing could reflect how to optimize Asian or Caucasian face processing [61]. Thus, even Caucasian children would do better to look centrally for Asian face processing and in specific areas in Caucasian faces. Also, the multicultural environment of the Swiss population could have made it possible to develop the emergence of these optimal strategies, while keeping an Other-Race Effect, which would be behaviorally invisible but psychophysically observable through the eye-tracker. Our study reveals that in an integrated multicultural environment, the behavioral emotional Other-Race Effect is not systematically found, suggesting that own-race bias can be hidden by various inter-ethnical experiences. Nevertheless, as suggested by Kelly and collaborators [62], even if the environment modulates expertise for type of faces experienced, this social experience seems not to abolish cultural diversity in eye movement strategies. Indeed, even though behavioral emotional ORE was not found in our sample, the eye-tracker revealed a silent ORE through ocular movements. This tool allows us to observe a developmental course in emotional processing, confirming an earlier understanding of happiness compared to anger, and shows longer looking time towards mouth area. Previous studies have suggested a Caucasian analytic strategy in face processing contrasted with an Asian ‘central’ holistic strategy [58]. From 7 year-old, children would already displayed patterns of fixations in internal features consistent with adults of their cultural groups [77]. Thus, our results could present above all a developmental period in which the gaze is oriented principally around the mouth due to abilities developed for happiness processing, during which children learn to balance their gaze between the eyes and mouth according to the emotion. Nevertheless, we highlight that even Caucasian children seem to process Asian faces, particularly the more complex anger emotion, with a more central or balanced strategy in order to find their cultural emotional cues. These results seem to inform about a flexible and crucial emotional developmental period, during which component and holistic strategies are evolving depending on expertise and familiarity to faces type to optimize performance. Nevertheless, the understanding of holistic *vs*. component strategies used depending on age or ethnicity requires major further investigation and specific studies, such as manipulating the inversion effect with eye-tracker recording, and fine gaze analysis with adapted stimuli. In conclusion, this study strengthens the interest to develop research on eye-tracking in developmental and emotional processing studies, showing that even a “silent” effect should be detected and shrewdly analyzed through an objective means.

## Supporting information

S1 Dataset Reaction times and looking times raw data. (XLSX)

## References

[1] Bayet L, Pascalis O, Gentaz E. The development of emotional facial expression discrimination by infants in the first year of life. Ann Psychol. 2014;114(3):469–500.

[2] Grossmann T. The development of emotion perception in face and voice during infancy. Restor Neurol Neurosci. 2010;28(2):219–36. Epub 2010/04/21. 10.3233/RNN-2010-0499 . 20404410

[3] Palama A, Malsert J, Gentaz E. Are 6-month-old human infants able to transfer emotional information (happy or angry) from voices to faces? An eye-tracking study. Plos One. 2018;13(4):e0194579 Epub 2018/04/12. 10.1371/journal.pone.0194579 . 29641530 PMC5894971

[4] Kolb B, Wilson B, Taylor L. Developmental changes in the recognition and comprehension of facial expression: implications for frontal lobe function. Brain Cogn. 1992;20(1):74–84. Epub 1992/09/01. 10.1016/0278-2626(92)90062-q . 1389123

[5] Gosselin P. Le décodage de l’expression faciale des émotions au cours de l’enfance. Canadian Psychology/Psychologie canadienne. 2005;46(3):126.

[6] Theurel A, Witt A, Malsert J, Lejeune F, Fiorentini C, Barisnikov K, et al The integration of visual context information in facial emotion recognition in 5-to 15-year-olds. J Exp Child Psychol. 2016;150:252–71. 10.1016/j.jecp.2016.06.004 27367301

[7] Widen SC, Russell JA. Young children’s understanding of other’s emotions. Handbook of emotions. 2008;3:348–63.

[8] Boyatzis CJ, Chazan E, Ting CZ. Preschool Childrens Decoding of Facial Emotions. J Genet Psychol. 1993;154(3):375–82. 10.1080/00221325.1993.10532190 8245911

[9] Camras LA, Allison K. Childrens Understanding of Emotional Facial Expressions and Verbal Labels. J Nonverbal Behav. 1985;9(2):84–94. 10.1007/Bf00987140

[10] Widen SC, Russell JA. Children’s recognition of disgust in others. Psychol Bull. 2013;139(2):271–99. Epub 2013/03/06. 10.1037/a0031640 . 23458434

[11] Balconi M, Carrera A. Emotional representation in facial expression and script A comparison between normal and autistic children. Res Dev Disabil. 2007;28(4):409–22. Epub 2006/07/11. 10.1016/j.ridd.2006.05.001 . 16828256

[12] Widen SC, Russell JA. The relative power of an emotion’s facial expression, label, and behavioral consequence to evoke preschoolers’ knowledge of its cause. Cognitive Dev. 2004;19(1):111–25. 10.1016/j.cogdev.2003.11.004

[13] Widen SC, Russell JA. Children’s scripts for social emotions: Causes and consequences are more central than are facial expressions. Brit J Dev Psychol. 2010;28(3):565–81. 10.1348/026151009X457550d 20849034

[14] Glasel H, Mazeau M. Chapitre 5—Évaluation des troubles gnosiques visuels In: Glasel H, Mazeau M, editors. Conduite du bilan neuropsychologique chez l’enfant (Troisième Édition). Paris: Elsevier Masson; 2017 p. 177–99.

[15] Meissner CA, Brigham JC. Thirty years of investigating the own-race bias in memory for faces—A meta-analytic review. Psychol Public Pol L. 2001;7(1):3–35. 10.1037//1076-8971.7.1.3

[16] Shepherd JW, Deregowski JB. Races and Faces—a Comparison of the Responses of Africans and Europeans to Faces of the Same and Different Races. Brit J Soc Psychol. 1981;20(Jun):125–33. 10.1111/j.2044-8309.1981.tb00485.x

[17] Bothwell RK, Brigham JC, Malpass RS. Cross-Racial Identification. Personality and Social Psychology Bulletin. 1989;15(1):19–25. 10.1177/0146167289151002

[18] Rhodes G, Brake S, Taylor K, Tan S. Expertise and Configural Coding in Face Recognition. Brit J Psychol. 1989;80:313–31. 10.1111/j.2044-8295.1989.tb02323.x 2790391

[19] Sangrigoli S, de Schonen S. Recognition of own-race and other-race faces by three-month-old infants. J Child Psychol Psyc. 2004;45(7):1219–27. 10.1111/j.1469-7610.2004.00319.x 15335342

[20] Sangrigoli S, Pallier C, Argenti AM, Ventureyra VAG, de Schonen S. Reversibility of the other-race effect in face recognition during childhood. Psychol Sci. 2005;16(6):440–4. 10.1111/j.0956-7976.2005.01554.x 15943669

[21] Ge LZ, Zhang HC, Wang Z, Quinn PC, Pascalis O, Kelly D, et al Two faces of the other-race effect: Recognition and categorisation of Caucasian and Chinese faces. Perception. 2009;38(8):1199–210. 10.1068/p6136 19817152

[22] Brigham JC, Malpass RS. The Role of Experience and Contact in the Recognition of Faces of Own-Race and Other-Race Persons. J Soc Issues. 1985;41(3):139–55. 10.1111/j.1540-4560.1985.tb01133.x

[23] Chiroro P, Valentine T. An Investigation of the Contact Hypothesis of the Own-Race Bias in Face Recognition. Q J Exp Psychol-A. 1995;48(4):879–94. 10.1080/14640749508401421

[24] Valentine T. A Unified Account of the Effects of Distinctiveness, Inversion, and Race in Face Recognition. Q J Exp Psychol-A. 1991;43(2):161–204. 10.1080/14640749108400966 1866456

[25] Kelly DJ, Quinn PC, Slater AM, Lee K, Gibson A, Smith M, et al Three-month-olds, but not newborns, prefer own-race faces. Developmental Sci. 2005;8(6):F31–F6. 10.1111/j.1467-7687.2005.0434a.x 16246233 PMC2566511

[26] Nelson CA. The development and neural bases of face recognition. Infant Child Dev. 2001;10(1–2):3–18. 10.1002/icd.239

[27] Pascalis O, de Haan M, Nelson CA. Is face processing species-specific during the first year of life? Science. 2002;296(5571):1321–3. 10.1126/science.1070223 12016317

[28] Kelly DJ, Liu SY, Ge LZ, Quinn PC, Slater AM, Lee K, et al Cross-race preferences for same-race faces extend beyond the African versus Caucasian contrast in 3-month-old infants. Infancy. 2007;11(1):87–95. 10.1080/15250000709336871 18974853 PMC2575407

[29] Pascalis O, Scott LS, Kelly DJ, Shannon RW, Nicholson E, Coleman M, et al Plasticity of face processing in infancy. Proc Natl Acad Sci U S A. 2005;102(14):5297–300. 10.1073/pnas.0406627102 15790676 PMC555965

[30] Hayden A, Bhatt RS, Joseph JE, Tanaka JW. The other-race effect in infancy: Evidence using a morphing technique. Infancy. 2007;12(1):95–104. 10.1111/j.1532-7078.2007.tb00235.x 33412731

[31] Kelly DJ, Quinn PC, Slater AM, Lee K, Ge LZ, Pascalis O. The other-race effect develops during infancy—Evidence of perceptual narrowing. Psychol Sci. 2007;18(12):1084–9. 10.1111/j.1467-9280.2007.02029.x 18031416 PMC2566514

[32] Hayden A, Bhatt RS, Zieber N, Kangas A. Race-based perceptual asymmetries underlying face processing in infancy. Psychon B Rev. 2009;16(2):270–5. 10.3758/Pbr.16.2.270 19293093 PMC7670847

[33] Pascalis O, Kelly DJ. The Origins of Face Processing in Humans: Phylogeny and Ontogeny. Perspect Psychol Sci. 2009;4(2):200–9. 10.1111/j.1745-6924.2009.01119.x 26158945

[34] Quinn PC, Yahr J, Kuhn A, Slater AM, Pascalis O. Representation of the gender of human faces by infants: A preference for female. Perception. 2002;31(9):1109–21. 10.1068/p3331 12375875

[35] Anzures G, Quinn PC, Pascalis O, Slater AM, Tanaka JW, Lee K. Developmental Origins of the Other-Race Effect. Curr Dir Psychol Sci. 2013;22(3):173–8. 10.1177/0963721412474459 24049246 PMC3773883

[36] Bar-Haim Y, Ziv T, Lamy D, Hodes RM. Nature and nurture in own-race face processing. Psychol Sci. 2006;17(2):159–63. 10.1111/j.1467-9280.2006.01679.x 16466424

[37] Heron-Delaney M, Anzures G, Herbert JS, Quinn PC, Slater AM, Tanaka JW, et al Perceptual Training Prevents the Emergence of the Other Race Effect during Infancy. Plos One. 2011;6(5). ARTN e19858 10.1371/journal.pone.0019858 21625638 PMC3097220

[38] Anzures G, Quinn PC, Pascalis O, Slater AM, Tanaka JW, Lee K. Developmental Origins of the Other-Race Effect. Curr Dir Psychol Sci. 2013;22(3):173–8. Epub 2013/09/21. 10.1177/0963721412474459 24049246 PMC3773883

[39] de Heering A, de Liedekerke C, Deboni M, Rossion B. The role of experience during childhood in shaping the other-race effect. Developmental Sci. 2010;13(1):181–7. 10.1111/j.1467-7687.2009.00876.x 20121874

[40] De Viviés X, Kelly DJ, Cordier V, Pascalis O. Reconnaissance des visages d’un autre groupe ethnique: éclairage d’une approche développementale. Psychol Fr. 2010;55(3):243–57.

[41] Carroo AW. Other Race Recognition—a Comparison of Black-American and African Subjects. Percept Mot Skills. 1986;62(1):135–8. 10.2466/pms.1986.62.1.135 3960654

[42] Carroo AW. Recognition of Faces as a Function of Race, Attitudes, and Reported Cross-Racial Friendships. Percept Mot Skills. 1987;64(1):319–25. 10.2466/pms.1987.64.1.319

[43] Kilbride JE, Yarczower M. Ethnic Bias in the Recognition of Facial Expressions. J Nonverbal Behav. 1983;8(1):27–41. 10.1007/Bf00986328

[44] Markham R, Wang L. Recognition of emotion by Chinese and Australian children. J Cross Cult Psychol. 1996;27(5):616–43. 10.1177/0022022196275008

[45] Elfenbein HA, Ambady N. Is there an in-group advantage in emotion recognition? Psychol Bull. 2002;128(2):243–9. Epub 2002/04/05. 10.1037/0033-2909.128.2.243 . 11931518

[46] Elfenbein HA, Mandal MK, Ambady N, Harizuka S, Kumar S. Cross-cultural patterns in emotion recognition: highlighting design and analytical techniques. Emotion. 2002;2(1):75–84. Epub 2003/08/06. 10.1037/1528-3542.2.1.75 . 12899367

[47] Safar K, Kusec A, Moulson MC. Face Experience and the Attentional Bias for Fearful Expressions in 6-and 9-Month-Old Infants. Frontiers in Psychology. 2017;8 ARTN 1575 10.3389/fpsyg.2017.01575 28979221 PMC5611515

[48] Elfenbein HA, Ambady N. On the universality and cultural specificity of emotion recognition: a meta-analysis. Psychol Bull. 2002;128(2):203–35. Epub 2002/04/05. 10.1037/0033-2909.128.2.203 . 11931516

[49] Matsumoto D. Methodological requirements to test a possible in-group advantage in judging emotions across cultures: Comment on Elfenbein and Ambady (2002). Psychol Bull. 2002;128(2):236–42. 10.1037/0033-2909.128.2.236 11931517

[50] Chen XY, Rubin KH, Liu MW, Chen HC, Wang L, Li D, et al Compliance in Chinese and Canadian toddlers: A cross-cultural study. Int J Behav Dev. 2003;27(5):428–36. 10.1080/01650250344000046

[51] Gartstein MA, Gonzalez C, Carranza JA, Ahadi SA, Ye RN, Rothbart MK, et al Studying cross-cultural differences in the development of infant temperament: People’s Republic of China, the United States of America, and Spain. Child Psychiat Hum D. 2006;37(2):145–61. 10.1007/s10578-006-0025-6 16874564

[52] Sabbagh MA, Xu F, Carlson SM, Moses LJ, Lee K. The development of executive functioning and theory of mind—A comparison of Chinese and US preschoolers. Psychol Sci. 2006;17(1):74–81. 10.1111/j.1467-9280.2005.01667.x 16371147 PMC2567057

[53] Ishii K, Miyamoto Y, Mayama K, Niedenthal PM. When Your Smile Fades Away: Cultural Differences in Sensitivity to the Disappearance of Smiles. Soc Psychol Pers Sci. 2011;2(5):516–22. 10.1177/1948550611399153

[54] Niedenthal PM, Brauer M, Halberstadt JB, Innes-Ker AH. When did her smile drop? Facial mimicry and the influences of emotional state on the detection of change in emotional expression. Cognition Emotion. 2001;15(6):853–64. 10.1080/02699930143000194

[55] Chen XY, Hastings PD, Rubin KH, Chen HC, Cen GZ, Stewart SL. Child-rearing attitudes and behavioral inhibition in Chinese and Canadian toddlers: A cross-cultural study. Dev Psychol. 1998;34(4):677–86. 10.1037//0012-1649.34.4.677 9681259

[56] Rubin KH, Hemphill SA, Chen XY, Hastings P, Sanson A, Lo Coco A, et al A cross-cultural study of behavioral inhibition in toddlers: East-West-North-South. Int J Behav Dev. 2006;30(3):219–26. 10.1177/0165025406066723

[57] Dollion N, Soussignan R, Durand K, Schaal B, Baudouin JY. Visual exploration and discrimination of emotional facial expressions in 3-, 7-and 12-month-old infants. Journal of vision. 2015;15(12):e795–e.

[58] Blais C, Jack RE, Scheepers C, Fiset D, Caldara R. Culture Shapes How We Look at Faces. Plos One. 2008;3(8). ARTN e3022 10.1371/journal.pone.0003022 18714387 PMC2515341

[59] Liu S, Quinn PC, Wheeler A, Xiao N, Ge L, Lee K. Similarity and difference in the processing of same- and other-race faces as revealed by eye tracking in 4- to 9-month-olds. J Exp Child Psychol. 2011;108(1):180–9. 10.1016/j.jecp.2010.06.008 20708745 PMC3740558

[60] Wheeler A, Anzures G, Quinn PC, Pascalis O, Omrin DS, Lee K. Caucasian infants scan own- and other-race faces differently. Plos One. 2011;6(4):e18621 Epub 2011/05/03. 10.1371/journal.pone.0018621 21533235 PMC3076379

[61] Kitayama S, Duffy S, Kawamura T, Larsen JT. Perceiving an object and its context in different cultures: A cultural look at New Look. Psychol Sci. 2003;14(3):201–6. 10.1111/1467-9280.02432 12741741

[62] Kelly DJ, Jack RE, Miellet S, De Luca E, Foreman K, Caldara R. Social experience does not abolish cultural diversity in eye movements. Frontiers in Psychology. 2011;2 ARTN 95 10.3389/fpsyg.2011.00095 21886626 PMC3154403

[63] Ekman P, Friesen WV. Facial action coding system: Investigator’s guide: Consulting Psychologists Press; 1978.

[64] Malsert J, Tran K, Thi Tran TA, Ha-Vinh T, Gentaz E, Ha-Vinh Leuchter R. Cross-cultural and environmental influences on facial emotional perception sensitivity in 9-year-old children from Swiss and Vietnamese schools. Under revison.

[65] Halberstadt JB, Niedenthal PM. Effects of emotion concepts on perceptual memory for emotional expressions. J Pers Soc Psychol. 2001;81(4):587–98. Epub 2001/10/20. . 11642347

[66] Korb S, Malsert J, Rochas V, Rihs TA, Rieger SW, Schwab S, et al Gender differences in the neural network of facial mimicry of smiles—An rTMS study. Cortex. 2015;70:101–14. 10.1016/j.cortex.2015.06.025 26211433

[67] Korb S, Malsert J, Strathearn L, Vuilleumier P, Niedenthal P. Sniff and mimic—Intranasal oxytocin increases facial mimicry in a sample of men. Horm Behav. 2016;84:64–74. 10.1016/j.yhbeh.2016.06.003 27283377

[68] Wong JJ, Cho SY. A local experts organization model with application to face emotion recognition. Expert Syst Appl. 2009;36(1):804–19. 10.1016/j.eswa.2007.10.030

[69] Wong JJ, Cho SY. A Brain-Inspired Model for Recognizing Human Emotional States from Facial Expression In: Perlovsky LI, Kozma R, editors. Neurodynamics of Cognition and Consciousness. Berlin, Heidelberg: Springer Berlin Heidelberg; 2007 p. 233–54.

[70] Berdasco-Muñoz E, Nazzi T, Yeung HH. Visual scanning of a talking face in preterm and full-term infants. Dev Psychol. 2019;55(7):1353–61. 10.1037/dev0000737 31070435

[71] Hutmacher W. L’école du village mondial. Le Globe Revue genevoise de géographie. 1995;135(1):53–61.

[72] Genève OCdSd. Bilan et état de la population du canton de Genève en 2017. Mars 2018 ed: Informations statistiques; 2018.

[73] Derntl B, Seidel E-M, Kainz E, Carbon C-C. Recognition of Emotional Expressions is Affected by Inversion and Presentation Time. Perception. 2009;38(12):1849–62. 10.1068/p6448 . 20192133

[74] Leppänen JM, Hietanen JK. Is there more in a happy face than just a big smile? Vis Cogn. 2007;15(4):468–90.

[75] Cassia VM, Picozzi M, Kuefner D, Bricolo E, Turati C. Holistic processing for faces and cars in preschool-aged children and adults: evidence from the composite effect. Developmental Sci. 2009;12(2):236–48. 10.1111/j.1467-7687.2008.00765.x 19143797

[76] Crookes K, McKone E. Early maturity of face recognition: no childhood development of holistic processing, novel face encoding, or face-space. Cognition. 2009;111(2):219–47. Epub 2009/03/20. 10.1016/j.cognition.2009.02.004 . 19296930

[77] Kelly DJ, Liu SY, Rodger H, Miellet S, Ge LZ, Caldara R. Developing cultural differences in face processing. Developmental Sci. 2011;14(5):1176–84. 10.1111/j.1467-7687.2011.01067.x 21884332

